# Central line bundle including split-septum device and single-use prefilled flushing syringes to prevent port-associated bloodstream infections: a cost and resource-utilization analysis

**DOI:** 10.1186/s12913-020-05221-6

**Published:** 2020-04-21

**Authors:** İlker Devrim, Mustafa Taha Özkul, İlknur Çağlar, Yeliz Oruç, Nevbahar Demiray, Neryal Tahta, Canan Vergin

**Affiliations:** 1 Department of Pediatric Infectious Diseases, Dr. Behçet Uz Children’s Hospital, İzmir, Turkey; 2 Department of Pediatrics, Dr. Behçet Uz Children’s Hospital İzmir, İzmir, Turkey; 3 Department of Infection Control Committee, Dr. Behcet Uz Children’s Hospital, Izmir, Turkey; 4 Department of Pediatric Hematology and Oncology, Dr. Behçet Uz Children’s Hospital, Izmir, Turkey

**Keywords:** Central line bundle, Cost-effectiveness, Pediatric malignancy, Port associated bloodstream infections

## Abstract

**Background:**

Central line bundle programs were found to be effective in decreasing central line-associated bloodstream infection rates in pediatric cancer patients with ports. However, cost-effectiveness studies of central line bundle programs in pediatric cancer patients are limited, and most available data are from intensive care unit or adult studies.

**Methods:**

In this cross-sectional study spanning 6 years, comprehensive assessment of total health care costs attributable to CLABSI’s associated with ports between two periods.

**Results:**

This cross-sectional study was carried out in the pediatric hematology-oncology ward of Dr. Behçet Uz Children’s Hospital from 1 August November 2011 to 31 July 2017. The CLABSI rates decreased significantly from 8.31 CLABSIs to 3.04 per 1000 central line days (*p* < 0.001). In the pre-bundle period, total attributable costs spent for of patients with CLABSI were $130,661, and in the bundle period, total attributable costs spent for patients with CLABSI were $116,579. Within bundle implantation, 71 potential CLABSI were prevented, which saved an additional $208,977.

**Conclusion:**

Our study shows that central line bundles decreases not only the CLABSI rate but also decreases attributable costs due to CLABSI. Expenses spent for bundle elements, were covered by savings by preventing CLABSI with higher costs.

## Background

Totally implantable venous access devices (TIVADs, ports) are one of the essential elements of cancer treatment and improve quality of life significantly [1]. Totally implantable venous access devices or implantable catheter ports are devices that can be implanted subcutaneously and enable continued or repeated access to the vascular system via veins. In a recent study from the United States, central line-associated blood stream infections (CLABSIs) associated with ports increased from 0.16 to 1.48 port days [2]. However, when they occur, they increase morbidity and mortality significantly [3, 4]. In a cohort study from a German paediatric oncology unit, including 43 paediatric cancer patients and 43 matched control patients, Biwersi et al. reported an additional attributable cost of $6970 and an increased length of stay of 12 days [5]. Another study from a tertiary centre in the Unites States reported an additional cost of $69,332 and an additional attributable length of stay of 21.2 days per CLABSI in paediatric haematology/oncology patients [6]. In the latter study, half of the patients with CLABSI had acute lymphoblastic leukaemia or acute myelogenous leukaemia [6].

Central line bundles (CLBs) for the prevention of CLABSIs include chlorhexidine gluconate skin preparations and complying with maximal sterile barriers during insertion, prefer the subclavian or internal jugular vein instead of the femoral vein, and require strict hand hygiene and daily review of necessity of central line. Central line bundles were reported to prevent CLABSI, especially in intensive care units [7–11]. Studies including CLB applications for long-term TIVADs in paediatric cancer patients have mainly focused on the impact on CLABSI rates in clinical settings, and to our knowledge, no available data are available in paediatric cancer patients [12–16].

In this cross-sectional study spanning 6 years, we performed a cost-effectiveness study for CLBs to prevent CLABSIs associated with TIVADs in paediatric cancer patients by reviewing our experience based on CLABSIs prevented by CLBs.

## Methods

### Setting

This cross-sectional study was carried out in the paediatric haematology-oncology ward of Dr. Behçet Uz Children’s Hospital in Turkey from 1 August 2011 to 31 July 2017. Dr. Behçet Uz Children’s Hospital is a 400-bed paediatric teaching hospital. At the time of the study, the oncology-haematology department had 28 beds and received 60 newly diagnosed haematologic and oncologic patients per year.

The study included 2 time periods: the 3-year baseline period before the initiation of CLB (1 Aug 2011 to 31 July 2014) and the 3-year period of CLB (1 Aug 2014 to 31 July 2017). The data were evaluated and recorded by three investigators, and the other investigators were blinded to the patients’ information to protect patient confidentiality.

According to our infection control committee policy, ports were the preferred route for vascular access for paediatric haematology-oncology patients and were inserted before starting induction chemotherapy under sterile conditions in the operating room.

#### Microbiology and definitions

In our centre, two central venous blood culture samples for aerobic and anaerobic cultures were taken from patients with fever under aseptic conditions and after disinfection of the central venous access device hub, in addition to a blood culture sample from peripheral veins. Fever was defined as a body temperature > 38.5 °C for at least 4 h or a body temperature > 39 °C once. Neutropenia was described as a total number of granulocytes < 0.5 × 10^9^/L or a total number of leukocytes < 1.0 × 10^9^/L without differential counts available [17].

Each blood culture bottle (taken from blood or ports) was placed in the BacT/ALERT 9240 automated system (bioMérieux, Marcy l’Etoile, France) [18]. The microorganisms were identified with a VITEK-2 compact system bioMérieux). Identification and antibiotic susceptibility tests of Gram-positive bacteria were performed using the automated VITEK-2 system with Gram-positive identification card ASTP592, a supplementary Etest (bio- Mérieux, Durham, NC, USA), and a disk diffusion test according to the manufacturer’s instructions [19]. This system was also used for the identification and antibiotic susceptibility tests of Gram-negative bacteria with Gram-negative identification cards AST-N325, AST-N326, and AST- N327 [20]. Yeast identification was performed using API 20 °C AUX (bioMérieux) [21].

#### Pre-bundle and bundle periods

The pre-bundle period included three years before the implementation of CLB. During this period, povidone-iodine at 10% was used for disinfection; three-way stopcocks were the choice of connection. In this period, flushing was performed using 5 or 10 mL of 0.9% NaCl, which was manually filled from everyday polyvinyl chloride bags.

The three-year CLB period comprised the following elements, including steps of needle insertion in the ports and management. The measures, including insertion of the needle in the ports, included strict hand hygiene, chlorhexidine skin antisepsis (> 0.5% chlorhexidine in an alcohol solution), and complying with maximal barrier precautions. The maintenance steps included daily inspection of the catheter sites and cap connection, disinfection of the hub with > 0.5% chlorhexidine in alcohol solution, use of needless connectors (BD Q-Syte; BD, Sandy, UT), use of sterile gauze or transparent semi-permeable membrane and use of single-use prefilled saline syringes (BD PosiFlush SP, BD, NJ) for flushing.

Employees were informed about bundle implementations, and feedback was made on bundle adjustment rates and CLABSI rates in addition to educational programmes. The educational programme was provided weekly in the first six months and monthly by the bundle team, which included an infection-control nurse and a paediatric infectious disease specialist. Educational sessions were provided to all healthcare workers in the haematology-oncology ward regarding the bundle implementations for CLABSI prevention described above. Compliance with the bundle was observed by supervisor nurses and recorded in the checklist forms. The study period, prospective and active surveillance for CLABSI rates, and the monitoring of compliance with the bundle components were performed by the bundle team weekly.

#### Cost-analysis and statistics

Demographic and clinical features with hospital costs and length of stay were retrospectively retrieved from the medical files and computerized hospital system. Direct medical care cost items were calculated with the hospital perspective using a combination of micro-costing technique (resource-based accounting method) and hospital list data. The direct medical costs mainly included charges for inpatient, laboratory, imaging, antimicrobial drugs, and other medications, plus operation costs for the removal of the ports if required. Attributable length of stay for hospital admission was considered as the span of days for the treatment of CLABSI. The investigators recorded the costs first in Turkish Lira (TL) and converted them to USD ($), using the average exchange rate between TL to USD currency between 1 August 2011 to 31 July 2017 (1TL = 0.4484 $) [22]. During the study, we did apply equal discounting of costs and health effects, which was supported by Weinstein and Stason’s consistency thesis [23] and “time neutrality” by Lipscomb et al. [24]. The investigators did track the costs and outcomes during the patients stay in the haematology-oncology department.

Descriptive analysis of the data was carried out by using the IBM SPSS Statistics 17.0 programme (IBM Corporation, Armonk, NY, USA). The numbers of CLABSI per day was calculated for each group. The rate of infections in two different populations was compared with the Poisson 95% confidence interval in each bundle group, and the relative risk reduction between groups was calculated and given as percentages. The relative risk reduction rate was also calculated to compare the risks for every two groups, with a 95% confidence interval for the incidence rate. The statistical analysis was performed using Medcalc v 11.6 (Ostend Belgium).

This study was approved by the local ethical committee and the institutional review board of Dr. Behçet Uz Children’s Training and Research Hospital.

## Results

During the six years of study period, the total number of catheter line days was 18,672 (Table 1). Total CL days were 5175 in the pre-bundle period and 13,497 days in the bundle period. During the pre-bundle period, there were 43 CLABSIs in 5175 CL-days with an overall rate of 8.31 CLABSIs per 1000 CL-days. After implantation of the central line bundles, there were 41 CLABSIs in 13,497 CL-days, which accounts for an overall rate of 3.04 CLABSIs per 1000 CL-days (Table 1). The incidence rate difference between these two groups was 0.005, indicating a relative risk reduction of 59% (*p* < 0.001; Table 1).

**Table 1:** The distribution of microorganisms associated with CLABSI’s associated with TIVADs

	Pre-Bundle period	Bundle period
Port days	5175	13,497
Central line days	43	41
CLABSI rate (per 1000 CL days)	8.31	3.04
Hospital stay costs	$14,744	$14,493
Drug costs	$73,231	$ 48,169
Laboratory costs	$34,974	$14,163
Imaging costs	$1122	$1037
Consultation costs	$535	$936
Other costs	$6053	$5835
Bundle item costs	–	$31,944
Total costs	$130,661	$116,579

Among the 84 CLABSIs, eight infections were poly-microbial (9.5%). Among the 92 isolations, 59 isolations were gram-negative bacteria (64.1%), followed by gram-positive bacteria (18.4%) and fungal infections (17.3%). The most common isolated microorganisms across the entire six-year period were *Klebsiella pneumoniae* (21.7%), *Candida* species (16.3%), and *Escherichia coli* (14.1%). Infections due to *coagulase-negative Staphylococcus* and *S.aureus* were 8.6 and 3.2%, respectively. The other microorganisms are listed in Table 2.

**Table 2:** The comparison of port days, central line days, CLABSI rate, hospital stay costs, drug costs, laboratory, and total costs

Microorganisms	Total ^a^
*Klebsiella pneumoniae ssp pneumoniae*	20(21.7%)
*Candida species*^*b*^	15(16.3%)
*Escherichia coli*	13(14.1%)
*Coagulase negative staphylococcus*^*c*^	8(8,6%)
*Enterobacter cloacae*	7(7.6%)
*Klebsiella oxytoca*	6(6.5%)
*Streptococcus species*^*d*^	4(4.3%)
*Acinetobacter baumanni complex*	4(4.3%)
*Staphyloccous auerus*	3(3.2%)
*Pseudomonas aeruginosa*	3(3.2%)
*Stenotrophomonas maltophilia*	3(3.2%)
*Enterococcus faecium*	2(2.1%)
*Pseudomonas putida*	2(2.1%)
*Enterobacter sakazakii*	1(1,0%)
*Trichosporan ashaii*	1(1,0%)
Total	92(100,0%)

^*a*^*There were 8 polymicrobial CLABSIs of 84 CLABSIs*

^*b*^*Candida isolations included 8 C.parapsilosis, 2 C.albicans, 2 C.tropicalis, 1 C.kefyr, 1 C.guilliermondii, 1 C.krusei*

^*c*^*Coagulase negative staphyloccus isolations included 5 S.hominis, and 3 S.epidermidis*

^*d*^*Streptococcus speices included 2 S.mitis, 1 S.sangius and 1 S.oralis*

### Economic analysis and projection

In the prebendal period, the total attributable costs for patients with CLABSI were $130,661, and in the bundle period, the total attributable costs for patients with CLABSI were $116,579 (Table 2). The costs for items used for the bundle, including needless connectors, transparent semi-permeable membrane, and single-use prefilled saline syringes for three years, were $31,944. The majority of the costs were drugs in both the pre-bundle and bundle period with totals of $73,231 and $48,169, respectively. The attributable cost per CLABSI, including both the pre-bundle and bundle period, was $2943.

Before bundle intervention, the CLABSI rate was 8.31 CLABSIs per 1000 CL-days. In our projection, if we did not perform any CLB intervention and assumed that the CLABSI rate did not change, our patients would have had 112 CLABSI infections for 13,497 CL days. Within bundle implantation, we observed only 41 CLABSIs, and CLB implantation prevented 71 CLABSIs from occurring. The total money saved with bundle implantation was $208,977 (calculated by cost per CLABSI X number of prevented CLABSIs).

## Discussion

In this study, significant differences in the CLABSI incidence rate and significant savings with CLB were observed. The CLABSI rate decreased from 8.31 CLABSIs to 3.04 per 1000 CL-days after implantation of the CLB, and we saved $208,977 by preventing CLABSIs. This study is one of the few studies providing an economic view of CLBs for ports in paediatric malignancy patients.

Rinke et al. reported a decrease from 2.25 to 1.79 CLABSIs per 1000 central line days in paediatric haematology patients, including high CL days of 14,059 [13]. In another study from Germany in which CLB ready-to-use sterile NaCl 0.9% syringes were used, a decrease in BSI was observed; however, in the study from Germany, Broviac catheters were inserted instead of ports [17]. In another study from Turkey, CLB, including needless connectors and single-use prefilled saline syringes, significantly decreased CLABSI rates from 14.5 to 2.63 per 1000 CL-days. In this study, there was a total of 3831 CL days, and the study duration was approximately two years [16]. These studies, in addition to our study, showed the clinical efficiency of CLB programmes for prevention of CLABSIs in TIVADs [13, 16, 17].

To our knowledge, this is the first study that has been designed to evaluate the economic aspects of CLB, including needless connectors and single-use prefilled saline syringes, for prevention of CLABSIs in paediatric malignancy patients. The CLABSI numbers decreased from 43 to 41 during the pre-bundle period, while catheter days tripled reflecting the device utilization rate (5175 CL-days versus 13,497 CL-days). In our study, more complicated CLB was performed, and according to our projection, 71 CLABSIs were prevented due to the implantation of CLB if the same CLABSI rates were observed. The money spent on the CLB programme in our institute, including needless connectors, single-use prefilled saline syringes, and transparent semi-permeable membranes, was $31,944 for three years. Rosenthal et al. compared the cost-effectiveness of needless connectors and single-use prefilled saline syringes versus 3-way stopcocks in a randomized clinical trial [25]. The use of needless connectors and single-use prefilled saline syringes in adult intensive care units saved $124 for each extra dollar invested in the CLB [25]. Despite the difference in the patient characteristics, clinical settings, and design, these two studies demonstrated the cost-effectiveness of the CLB, including needless connectors and single-use prefilled saline syringes.

The cost calculations in our study were attributed to drugs, biochemical tests, blood culture, and radiological investigations to check whether they were directly or indirectly linked to the treatment of CLABSI. Thus, it reflected the correct attributable costs of CLABSI. Most of the cost was due to drugs (mostly antimicrobial costs) in both periods, but drug costs decreased during the bundle period in addition to laboratory costs. However, the saving was more significant when analysis included central line days. As central line days increase, the risk of developing infection also increases if precautions are not taken. Rosenthal et al. also calculated quality-adjusted life years and found an increase of 0.0008 quality-adjusted life years per patient [25]. In our study, we did not focus on the quality of life measures. However, a previous study from our clinic focusing on paediatric cancer patients reported that patients’ quality of life was improved by 90% due to prevention of port removal due to infections [16].

This study has limitations due to its design. First, data including costs were collected retrospectively from medical files, hospital accounting records, and data systems, and attributable costs were reviewed by two clinicians, which may have resulted in bias. Moreover, the current study mainly focused on measurable costs of CLABSI, and some other benefits and indirect savings of prevention of CLABSI could not be measured. For example, the time of delay in the treatment of primary disease was not measured. In the bundle period, flushing with the single-use prefilled saline syringes device, which is a 4-step process, compared to flushing using multi-dose vials, which is a 10-step process, was preferred, and the time saved by the nursing staff was not calculated. Moreover, neither sensitivity testing nor an analytical decision-making tree was applied, since we reviewed our real-life experience about CLB for preventing CLABSI.

## Conclusion

In our study, we found that the CLB programme, including needless connectors and single-use prefilled saline syringes devices, was associated with a significant clinical impact by lowering the CLABSI rate. Even though the study was retrospective, it yielded information on the economic perspective of preventing CLABSI with the CLBs.

## Data Availability

The datasets generated and/or analysed during the current study are not publicly available due [due to the policy of our hospital] but are available from the corresponding author on reasonable request.

## Abbrevations

CLABSI: Central Line-Associated Blood Stream Infections
TIVAD: Totally implantable venous access devices
CLB: Central line bundle
