# Correction to: Central line bundle including split-septum device and single-use prefilled flushing syringes to prevent port-associated bloodstream infections: a cost and resource-utilization analysis

**DOI:** 10.1186/s12913-020-05299-y

**Published:** 2020-05-21

**Authors:** İlker Devrim, Mustafa Taha Özkul, İlknur Çağlar, Yeliz Oruç, Nevbahar Demiray, Neryal Tahta, Canan Vergin

**Affiliations:** 1 Department of Pediatric Infectious Diseases, Dr. Behçet Uz Children’s Hospital, İzmir, Turkey; 2 Department of Pediatrics, Dr. Behçet Uz Children’s Hospital İzmir, İzmir, Turkey; 3 Department of Infection Control Committee, Dr. Behcet Uz Children’s Hospital, Izmir, Turkey; 4 Department of Pediatric Hematology and Oncology, Dr. Behçet Uz Children’s Hospital, Izmir, Turkey

**Correction to: BMC Health Serv Res 20, 336 (2020)**

**https://doi.org/10.1186/s12913-020-05221-6**

Following publication of the original article [1], the authors would like to exchange the Table 1’s caption and Table 2’s caption. The correct ones are shown below.

The sentence of Table 1’s caption currently reads:

**The comparison of port days, central line days, CLABSI rate, hospital stay costs, drug costs, laboratory, and total costs**

The sentence of Table 2’s caption currently reads:

**The distribution of microorganisms associated with CLABSI’s associated with TIVADs**
